# P-1180. Nuanced NICUs: developing neonatal intensive care unit infection prevention and control training for health departments

**DOI:** 10.1093/ofid/ofae631.1366

**Published:** 2025-01-29

**Authors:** Christopher Prestel, Katelyn White, Alicia Shugart, Austin R Penna, Raven Weems, Lauren Weil, David Kuhar, Ronda Sinkowitz-Cochran

**Affiliations:** Centers for Disease Control and Prevention, Atlanta, Georgia; Centers for Disease Control and Prevention, Atlanta, Georgia; Centers for Disease Control and Prevention, Atlanta, Georgia; Centers for Disease Control and Prevention, Atlanta, Georgia; Centers for Disease Control and Prevention, Atlanta, Georgia; Centers fo Disease Control and Prevention; Centers for Disease Control and Prevention, Atlanta, Georgia

## Abstract

**Background:**

Neonatal intensive care units (NICUs) provide life-sustaining care to premature neonates and infants with complex medical needs. NICU patients are at high risk for healthcare-associated infections (HAIs) and require nuanced infection prevention and control (IPC) strategies due to unique care requirements and medical equipment. Health departments are key in providing IPC technical assistance to NICUs for outbreak response and quality improvement initiatives; however, many health department personnel are unfamiliar with NICU settings. We created and delivered NICU-specific IPC training to help health departments improve their capacity to support NICUs.

**Methods:**

CDC developed three NICU-specific IPC educational webinars for health department personnel covering NICU settings, care, neonatal physiology, IPC practices and their nuances, and select NICU outbreak case reports. Health department personnel submitted demographic information and registered for the course electronically. Participants voluntarily responded to polls during webinars; questions included level of comfort, job duties, and NICU IPC concerns.

**Results:**

A total of 562 individuals enrolled in the course with 469 (84%) representing 59/64 (92%) of CDC-funded health department jurisdictions across the U.S.; most enrollees (59%) currently work in a non-IPC role (e.g., epidemiologist, program lead, data analyst). Most had five years or less time in their current role (84%), including 18% with less than a year of experience; 47% reported never providing technical assistance to a NICU in the past. Among poll participants (55%) reported being unaware of how many NICUs are in their jurisdiction. HAI risk associated with medical devices (46%), hand hygiene (20%), and environmental (19%) issues were perceived as the greatest IPC concerns in the NICU. After two of three training sessions, 94% of participants felt more confident providing IPC support to a NICU.

**Conclusion:**

Educational and training courses like these are an efficient way to improve health department capacity in providing IPC technical assistance to NICUs, and can help to build partnerships with healthcare facilities by strengthening infection prevention and quality improvement initiatives.

**Disclosures:**

**All Authors**: No reported disclosures

